# Comorbid Anxiety and Depression in Hyperacusis and Misophonia. Reply to Jastreboff, P.J. Comment on “Rodrigues, A.L.M.; Aazh, H. Psychiatric Comorbidities in Hyperacusis and Misophonia: A Systematic Review. *Audiol. Res*. 2025, *15*, 101”

**DOI:** 10.3390/audiolres15060147

**Published:** 2025-10-31

**Authors:** Hashir Aazh, Ana Luísa Moura Rodrigues

**Affiliations:** 1 Hashir International Specialist Clinics & Research Institute for Misophonia, Tinnitus and Hyperacusis, London W1W 5PF, UK; 2 Hospital das Forças Armadas, 1649-020 Lisboa, Portugal

This systematic review found that depression was reported in 8–80% of patients with hyperacusis and 1.1–37.3% of those with misophonia, while anxiety ranged from 39 to 61% in hyperacusis and 0.2 to 69% in misophonia [1]. These wide ranges reflect substantial methodological variability, much of it linked to reliance on self-report measures. As Jastreboff (2025) [2] observed, such methods may inflate prevalence estimates. Structured psychiatric interviews remain the most accurate approach to diagnosing depression and anxiety [3], yet most large-scale studies have not used them. This limitation carried forward into our review. Future research should combine psychiatric interviews with questionnaires, at least in subsamples, and apply statistical methods to improve prevalence precision.

Jastreboff (2025) [2] further remarked that self-report questionnaires fail to distinguish between situation-evoked or reactive depression, which arises in response to a stressor, and endogenous depression, which develops independently of external stress. In hyperacusis and misophonia, these conditions often act as the stressors, suggesting that depressive symptoms are more likely reactive. He also argued that depression and anxiety are too often interpreted as evidence of a primary “mental disorder,” without regard for underlying causes. By this logic, depression or anxiety secondary to illnesses such as cancer would also be considered primary psychiatric disorders, a problematic conclusion.

We agree that distinguishing between reactive and endogenous depression has treatment implications for patients with hyperacusis and misophonia. When reactive depression is identified through careful diagnostic interviewing, evidence supports brief stressor-focused approaches such as psychoeducation, problem-solving, and targeted CBT, with medication used selectively in collaboration between audiologists and mental health professionals. Without this distinction, prevalence may be inflated and sensory-intolerance conditions risk over-psychiatricization, with consequences for stigma, resource allocation, and research validity. Future studies should report the timing of symptom onset relative to sound intolerance and examine biological and neural markers of adjustment-related versus chronic psychiatric comorbidity. Greater diagnostic precision will improve prevalence estimates, refine clinical pathways, and enable more personalized care.

This is, however, a complex suggestion, as little research has examined depression subtypes in this population. The first point is that reactive depression or adjustment disorder is itself classified as a psychiatric illness, regardless of the precipitating stressor. Adjustment disorder is defined as a maladaptive response to identifiable psychosocial stressors such as illness, disability, socio-economic hardship, or interpersonal conflict [4], all of which could plausibly result from misophonia or hyperacusis. Typically emerging within a month of the stressor, adjustment disorder remains a diagnosable mental disorder. Therefore, distinguishing between reactive and endogenous depression may not necessarily reduce the proportion of patients with comorbid psychiatric conditions among those with hyperacusis or misophonia.

The second point is that depression associated with physical illness still requires treatment. For example, the risk of clinical depression among patients with cancer during the first year after diagnosis is 15–20% [5], and psychotherapy has been shown to improve mental health outcomes during or after treatment [6,7]. Thus, regardless of whether depression is primary or secondary, it warrants clinical intervention.

The third point is that not all patients with hyperacusis or misophonia experience anxiety or depression. In our review, depression was absent in 20–92% of patients with hyperacusis and 62.7–98.9% of those with misophonia. Anxiety was absent in 39–61% of patients with hyperacusis and 31–99.8% of those with misophonia [1]. This indicates that misophonia and hyperacusis are not simply manifestations of anxiety or depression but can occur independently of them.

The fourth point is that audiologists are the key professionals supporting patients with misophonia and hyperacusis. The term misophonia itself was coined by Jastreboff and Jastreboff in the context of their work on tinnitus and decreased sound tolerance retraining therapy [8]. In this role, audiologists must screen for symptoms of mental illness and refer patients to mental health services where appropriate. Our review highlights the importance of this collaborative model.

The fifth point is that developmental vulnerability and biological divergence complicate the “reactive versus endogenous” distinction. Early-life stress, including adverse childhood experiences (ACEs), is a well-established risk factor for adult depression through mechanisms such as stress sensitization, epigenetic change, and long-term alteration of stress response systems [9]. If hyperacusis or misophonia begin in childhood, or contribute to ACEs through bullying, social withdrawal, family conflict, or academic disruption, then depression later in adulthood may reflect a stress-sensitized phenotype rather than a straightforward adjustment to a current stressor. This makes the classification of such depression as purely “reactive” problematic.

## Conclusions

In summary, while Jastreboff (2025) [2] rightly highlights methodological limitations and the importance of distinguishing between depressive subtypes, the reality is more complex. Adjustment disorder remains a psychiatric diagnosis, depression associated with physical illness still requires treatment, and many patients with misophonia or hyperacusis do not present with psychiatric comorbidities. Audiologists therefore play a central role in screening and referral, ensuring that mental health needs are neither overlooked nor overstated. Finally, developmental vulnerability and biological divergence complicate a simple “reactive versus endogenous” classification. Future research must employ rigorous diagnostic methods, consider developmental context, and explore biological markers to refine prevalence estimates and clinical pathways. Such work will reduce stigma, improve care, and promote a more accurate understanding of the relationship between sensory intolerance and mental health.
